# Phase I study of cisplatin analogue nedaplatin (254-S) and paclitaxel in patients with unresectable squamous cell carcinoma

**DOI:** 10.1038/sj.bjc.6601700

**Published:** 2004-03-02

**Authors:** I Sekine, H Nokihara, A Horiike, N Yamamoto, H Kunitoh, Y Ohe, T Tamura, T Kodama, N Saijo

**Affiliations:** 1 Divisions of Internal Medicine and Thoracic Oncology, National Cancer Center Hospital, Tsukiji 5-1-1, Chuo-ku, Tokyo 104-0045, Japan

**Keywords:** squamous cell carcinoma, paclitaxel, nedaplatin, lung cancer

## Abstract

The recommended phase II dose of paclitaxel 180 mg m^−2^ given as a 3-h infusion followed by nedaplatin 100 mg m^−2^ in a 1-h infusion every 3–4 weeks was determined in 52 chemo-naive patients with unresectable squamous cell carcinoma (SCC), with a promising response rate for lung SCC of 55%.

Squamous cell carcinoma (SCC) arises from the epithelial tissue of many different organs. Although localised diseases can be treated using surgical resection or curative radiotherapy, advanced SCC continues to have a poor prognosis and the standard treatment has not been established (DeVita *et al*, 2001). Cisplatin-based chemotherapy has been used for the treatment of advanced SCC, regardless of the site of tumour origin (DeVita *et al*, 2001).

Nedaplatin (cis-diammine-glycolate-*O,O′*-platinum II, 254-S) is a second-generation platinum derivative that has an antitumour activity comparable to that of cisplatin (Kobayashi *et al*, 1991) but is less toxic to the kidney (Kameyama *et al*, 1990), as seen in preclinical experiments. Nedaplatin produced promising response rates in phase II trials for the treatment of SCC arising from the head and neck (Inuyama *et al*, 1992), lung (Yamamoto *et al*, 2000), oesophagus (Taguchi *et al*, 1992), and uterine cervix (Noda *et al*, 1992). Paclitaxel is another promising drug for the treatment of advanced SCC, as shown by the favourable response rates obtained in phase II trials for head and neck (Forastiere *et al*, 1998), non-small-cell lung (Sekine *et al*, 1996), oesophageal (Ajani *et al*, 1994), and cervical (McGuire *et al*, 1996) cancers.

A combination of nedaplatin and paclitaxel is a promising chemotherapeutic regimen because a significant synergistic effect was obtained for this combination in a preclinical mice tumour model (Yamada *et al*, 2001), and the combination of platinum compounds and paclitaxel is one of many standard regimens (Schiller *et al*, 2002). The objectives of this phase I trial were (1) to evaluate the toxicity of the regimen and to determine the maximum tolerated dose (MTD) and recommended phase II dose (RPTD) of nedaplatin and paclitaxel, and (2) to observe the antitumour effects of this regimen on SCC arising in various organs.

## PATIENTS AND METHODS

### Patient selection

The eligibility criteria for enrolment in the trial were as follows: histologically or cytologically proven SCC; unresectable disease; measurable disease; no previous chemotherapy; age between 20 and 75 years; performance status of 0 or 1 (Oken *et al*, 1982); adequate bone marrow function (white blood cell (WBC) count ⩾4.0 × 10^9^ l^−1^, neutrophil count ⩾2.0 × 10^9^ l^−1^, haemoglobin ⩾10.0 g dl^−1^ and platelet count ⩾100 × 10^9^ l^−1^), liver function (total bilirubin ⩽1.5 mg dl^−1^ and transaminase ⩽100 IU l^−1^), and renal function (serum creatinine ⩽1.5 mg dl^−1^ and creatinine clearance ⩾60 ml min^−1^); and a PaO_2_ ⩾60 Torr. Patients were excluded from the trial for any of the following reasons: uncontrolled malignant pleural or pericardial effusion; a concomitant serious illness contraindicating chemotherapy; pregnancy; or breast-feeding. All patients gave their written informed consent.

### Treatment schedule

The levels and respective doses of paclitaxel (mg m^−2^) and nedaplatin (mg m^−2^) are shown in Table 1

**Table 1:** Dose level and number of patients accrued

			**No. of patients**		
**Level**	**Paclitaxel (mg m^−2^)**	**Nedaplatin (mg m^−2^)**	**Accrued**	**Evaluable for DLT^a^**	**Developing DLT^a^**
1	135	60	6	6	2
2	150	60	3	3	0
3	150	80	3	3	0
4	180	80	7	6	1
5	180	100	12	12	4
6	210	100	21	19	8

a Dose-limiting toxicity.

. Paclitaxel diluted in 500 ml of 5% glucose was administered as a 3-h intravenous infusion with premedication as previously described (Sekine *et al*, 1996). Normal saline (500 ml) and granisetron (40 *μ*g kg^−1^) in 100 ml of normal saline were given intravenously, followed by nedaplatin diluted in 250 ml of normal saline administered in a 1-h intravenous infusion. This treatment was repeated every 3–4 weeks.

### Toxicity assessment and treatment modification

Complete blood cell counts and differential counts, routine chemistry determinations, and a chest X-ray were performed at least once a week throughout the course of treatment. If grade 4 neutropenia was noted, the neutrophil count was repeated 4 days later to determine whether the grade 4 neutropenia had lasted for 5 days or longer. Acute toxicity was graded according to the NCI Common Toxicity Criteria, version 2.0, issued in 1998 (JCOG, 1998). Subsequent cycles of chemotherapy were delayed if any of the following toxicities were noted on day 1: WBC count ⩽3.0 × 10^9^ l^−1^, neutrophil count ⩽1.5 × 10^9^ l^−1^, platelet count ⩽100 × 10^9^ l^−1^, serum creatinine level ⩾1.6 mg dl^−1^, grade 2 elevated hepatic transaminase level or total serum bilirubin, fever ⩾38°C, or a performance status ⩾2. The treatment was terminated if the above-mentioned toxicity did not disappear in 3 weeks. If grade 4 leukopenia, grade 4 neutropenia for 5 days or longer, grade 3–4 febrile neutropenia, or grade 3–4 neutropenia with infection was noted, 50 mg m^−2^ of granulocyte colony-stimulating factor (G-CSF) was given subcutaneously, and the doses of paclitaxel and nedaplatin were reduced by 25% in subsequent chemotherapy cycles.

### Dose-limiting toxicity, MTD, and RPTD

The dose-limiting toxicity (DLT) was defined as grade 4 neutropenia lasting 5 days or longer, grade 3–4 febrile neutropenia, grade 3–4 neutropenia with infection, grade 4 leukopenia, a platelet count <20 × 10^9^ l^−1^, and grade 3 or greater nonhaematological toxicity other than nausea and vomiting. Doses were escalated according to the frequency of DLT evaluated during the first cycle of chemotherapy. Three patients were initially enrolled at each dose level. If none of the patients experienced DLT, the next cohort of patients was treated at the next higher dose level. If one of the three patients experienced DLT, then three additional patients were enrolled at the same dose level, bringing the total to six patients for that dose level. If two or fewer patients experienced DLT, the next cohort of patients was treated at the next higher dose level. If three or more of the six patients experienced DLT, that level was considered to be the MTD. If two or all the initial three patients experienced DLT, that level was considered to be the MTD. The recommended dose for phase II trials was defined as the dose preceding the MTD. Six to 15 additional patients were enrolled at the RPTD to confirm that the frequency of DLT was less than one-third.

### Response evaluation

The objective tumour response was evaluated according to the WHO criteria issued in 1979 (WHO, 1979).

### Study design, data management, and statistical considerations

The protocol and consent form were approved by the Institutional Review Board of the National Cancer Center, Tokyo Japan. Data management, periodic monitoring, and the final analysis were performed by the Study Coordinator. A patient accrual period of 24 months and a follow-up period of 12 months were planned. The overall survival time was estimated using the Kaplan–Meier method (Armitage and Berry, 1994). Survival time was measured from the date of study registration until the date of death from any cause.

## RESULTS

### Patient characteristics

Between August 1999 and December 2002, 53 patients were registered in the study. One patient at level 5 developed a bone fracture prior to treatment and did not receive chemotherapy. This patient was excluded from all the analyses. Of the remaining 52 patients (42 males and 10 females) with a median age of 62 years (range 49–75), 42 (81%) patients had lung SCC, followed by thymic SCC in five patients and head and neck SCC in four patients. Of the 52 patients, 24 and 24 had metastatic and locally advanced diseases, respectively.

### Treatment delivery, toxicity, MTD, and RPTD

Treatment delivery was summarised in Table 2

**Table 2:** Treatment delivery

	**No. of patients (%)**		
	**Levels 1–4 (*n*=19)**	**Level 5 (*n*=12)**	**Level 6 (*n*=21)**
*Chemotherapy cycles*			
5	1 (5)	0 (0)	0 (0)
4	7 (37)	4 (33)	5 (24)
3	2 (11)	2 (17)	3 (14)
2	5 (26)	4 (33)	8 (38)
1	4 (21)	2 (17)	5 (24)
Median	3	3	2

*Dose reduction in subsequent cycles*			
None	12 (63)	9 (75)	12 (50)
Required	3 (16)	1 (8)	4 (19)
Not administered	4 (21)	2 (17)	5 (24)

. Severe toxicity was mainly manifested as leucopenia, neutropenia, and associated infection, but the frequency of these symptoms did not differ between dose levels (Table 3

**Table 3:** Toxicity in all courses

	**Levels 1–4 (*n*=19)**			**Level 5 (*n*=12)**			**Level 6 (*n*=21)**		
	**3**	**4**	**3–4 (%)**	**3**	**4**	**3–4 (%)**	**3**	**4**	**3–4 (%)**
Leukopenia	6	0	(32)	5	0	(42)	6	1	(33)
Neutropenia	3	10	(68)	2	9	(92)	3	12	(71)
Anaemia	0	0	(0)	0	0	(0)	1	0	(5)
Thrombocytopenia	0	0	(0)	0	0	(0)	1	0	(5)
AST	0	0	(0)	0	0	(0)	1	0	(5)
ALT	0	0	(0)	1	0	(8)	0	1	(5)
Creatinine	0	0	(0)	0	0	(0)	0	1	(5)
Hyponatremia	0	0	(0)	0	0	(0)	2	1	(14)
Infection	4	0	(21)	4	0	(33)	6	0	(29)
Appetite loss	0	0	(0)	0	0	(0)	1	0	(5)
Diarrhoea	0	0	(0)	0	0	(0)	2	0	(10)
Constipation	0	0	(0)	0	0	(0)	0	1	(5)
Arrhythmia	2	0	(11)	0	0	(0)	0	0	(0)
Lung toxicity	0	0	(0)	0	0	(0)	2	0	(10)

). Grade 3 anaemia and thrombocytopenia were only noted in one patient (5%) each; both these patients had been treated at dose level 6. No grade 3–4 nausea, neuropathy, or myalgia was noted. A grade 3–4 elevation in creatinine, grade 3–4 hyponatremia, appetite loss, and diarrhoea were only observed at level 6. One patient treated at level 6 developed grade 2 leukopenia, fever, watery diarrhoea, and grade 4 ileus, but recovered in 5 days. Two patients at level 6 developed grade 3 interstitial pneumonitis, but quickly recovered with oxygen therapy alone in one patient and with oxygen and steroid therapy in the other patient. No treatment-related deaths occurred in the study.

In all, 19 DLTs were noted in 15 patients. Of the 1nine DLTs, 13 were neutropenic fever or documented infection and six were nonhaematological. At level 6, only two of the first six patients developed DLT; therefore, 15 additional patients were entered at this level to confirm the frequency of DLT. Two patients were excluded from the DLT analysis because G-CSF was administered before the duration of grade 4 neutropenia had been determined (protocol violation). Of the remaining 13 patients, six developed DLT. Thus, eight (42%) of the 19 patients evaluated for DLT developed DLT at level 6; this dose level was therefore determined to be the MTD. An additional six patients were registered at level 5, and four (33%) of the 12 patients at level 5 developed DLT; this level was determined to be the RPTD.

### Objective responses and survival

Of the 42 patients with lung SCC, two CRs and 21 PRs were noted, and the overall response rate (95% confidence interval) was 55% (39–70%). No difference in the response rates for levels 1–4 and levels 5–6 were observed. One PR was noted in a patient with thymic SCC, and one PR was noted in a patient with head and neck SCC. The overall survival time (95% confidence interval) in all patients (*n*=52) was 11.1 (6.4–15.8) months.

## DISCUSSION

This study showed that the combination of nedaplatin and paclitaxel was feasible with acceptable toxicity, and that the RPTD of nedaplatin was 100 mg m^−2^ over 1 hour, which is the full dose of this agent, while that of paclitaxel was 180 mg m^−2^ over 3 h. These doses are comparable to doses for practical use and those determined by previous phase I trials of cisplatin or carboplatin in combination with paclitaxel, where 180–225 mg m^−2^ of paclitaxel was given with the full dose of platinum-agent (Akiyama *et al*, 2001; Kurata *et al*, 2001). The toxicity profile in the present study was similar to that of the carboplatin and paclitaxel combination (Akiyama *et al*, 2001).

The primary objectives of phase I trials are to evaluate toxicity and to establish a recommended drug dose for a given administration schedule; an additional goal of these trials is to look for evidence of the drug's antitumour activity. Objective tumour responses to newly investigated drugs are a promising clue for determining specific tumour types for subsequent phase II trials; therefore, patients with various tumours are usually registered in phase I trials (Sekine *et al*, 2002). In cases where some information on the antitumour activity of a drug is available, patients can be selected so that the chance of a response is maximised. This study was a histology-oriented phase I trial, and objective tumour responses were observed in about half of the patients.

The combination of nedaplatin and paclitaxel is particularly promising for the treatment of patients with lung SCC, as shown by the high response rate of 55%. Adenocarcinoma, large-cell carcinoma, adenosquamous carcinoma, and SCC of the lung have been grouped together as non-small-cell lung cancer because treatment response and prognosis are similar for these histologies. A recent cDNA microarray analysis of non-small-cell lung cancer tissue, however, showed that the gene expression profiles of SCC and adenocarcinoma are different (Kikuchi *et al*, 2003), and these differences may lead to different responses to anticancer agents, including nedaplatin. Thus, optimal chemotherapy regimens for the treatment of non-small-cell lung cancer should be established according to each tumour's histology. The numbers of patients with head and neck SCC and patients with thymic SCC were too small to comment on the antitumour effects of this regimen.

In conclusion, the combination of nedaplatin and paclitaxel is a feasible treatment, and the RPTD is paclitaxel 180 mg m^−2^ given as a 3-h infusion followed by nedaplatin 100 mg m^−2^ in a 1-h infusion every 3–4 weeks. This regimen was highly effective for the treatment of untreated lung SCC.
